# Active Craniospinal Tensioning (ACT) for Posture Correction and Spinal Decompression: Biomechanical Rationale for Glymphatic Clearance and Cerebral Venous Preconditioning (CVPC)

**DOI:** 10.7759/cureus.112433

**Published:** 2026-07-10

**Authors:** Huan-Wei Chen

**Affiliations:** 1 Chiropractic, Private Chiropractic Practice, Vancouver, CAN

**Keywords:** active craniospinal tensioning (act), alzheimer's disease, cerebral venous preconditioning (cvpc), diffusion tensor image analysis along the perivascular space (dti-alps), glymphatic system, neurodegenerative disease, posture correction, remote ischemic preconditioning (ripc), spinal decompression, spinal degenerative disease

## Abstract

For approximately two-thirds of each 24-hour period, the upright human spine bears a continuous gravitational load; yet no common movement or exercise naturally produces meaningful axial decompression of the entire spine. Active craniospinal tensioning (ACT) addresses this unmet need through a brief, self-administered squat maneuver against an overhead anchor, intended to generate reproducible axial spinal decompression without specialized equipment.

This report, which extends a previously published series on axial spinal traction, presents ACT as a postural correction and spinal decompression intervention. Building on those findings, a second distinct hypothesis is advanced: that two transient physiological events occurring simultaneously during the maneuver may together acutely accelerate glymphatic clearance. The first is a cerebrospinal fluid (CSF) pressure gradient generated during traction and rapidly reversed upon release, as detailed in the previous technical reports; the second is suboccipital venous occlusion during the tensioning phase, followed by a rebound upon release. The second mechanism, suboccipital venous occlusion-rebound, is further proposed as a novel venous-side modality for cerebral venous preconditioning (CVPC), distinct from existing arterial-based ischemic preconditioning approaches, whether local or remote (e.g., remote ischemic preconditioning (RIPC)).

Diffusion tensor image analysis along the perivascular space (DTI-ALPS) is proposed as the primary falsifiable endpoint for the glymphatic clearance hypothesis, which does not itself test the separately proposed CVPC effect of suboccipital venous occlusion-rebound. If confirmed, this endpoint would support glymphatic clearance as an additional mechanism of ACT, positioning ACT, alongside its proposed roles in posture correction, spinal decompression, and cerebral venous preconditioning (CVPC), as a brief, self-administered maneuver addressing multiple physiological targets through daily applications totaling less than one minute, without specialized equipment or clinical access.

## Introduction

The spine is an axially loaded structure continuously compressed by gravity throughout waking life. The head, weighing approximately 4.9 kilograms in adults, is the major contributor to cervical axial load. In neutral alignment, this weight is efficiently distributed along the vertical axis. In forward head posture, increasingly prevalent in sedentary populations, the effective cervical load rises substantially due to the increased lever arm. Finite element analysis shows that a 15-degree forward tilt adds approximately 7.2 kilograms of load and a 30-degree tilt adds roughly 13.1 kilograms, both compared with neutral posture [1]. Over time, this persistent compression contributes to intervertebral disc degeneration, facet joint overloading, paraspinal muscle tension, and progressive postural deterioration. The resulting low back pain and neck pain are among the most common musculoskeletal presentations in clinical practice [2].

Spinal movements primarily redistribute load asymmetrically rather than achieve uniform whole-disc decompression: lateral flexion compresses the ipsilateral annulus, while flexion-extension shifts intradiscal pressure anteriorly or posteriorly without reducing total axial load. In contrast, active craniospinal tensioning (ACT), a self-administered technique introduced in a prior technical report [3], is intended to achieve true whole-spine axial decompression. By applying craniocaudal tensile forces, ACT elongates the intervertebral discs along the spinal axis, a mechanism proposed as the principal means by which axial load may be reduced and postural alignment restored.

The present report extends the previously published ACT technical report [3] in three respects: the identification of posture correction and spinal decompression as primary structural endpoints, the reframing of suboccipital venous occlusion-rebound from a secondary effect warranting caution into a distinct therapeutic mechanism, and the formal development of diffusion tensor image analysis along the perivascular space (DTI-ALPS) [4], previously proposed in that report as a candidate surrogate biomarker for glymphatic function, into a structured falsifiable endpoint applicable across both single-session and longitudinal study designs. To address these extensions, an expanded biomechanical model is presented that incorporates two components: the dural pull-recoil mechanism, specifically the cerebrospinal fluid (CSF) pressure gradient generated during traction and rapidly reversed upon release, as detailed in the previous technical reports [3,5]; and venous occlusion-rebound dynamics, linking the biomechanical effects of ACT to glymphatic clearance. It is hypothesized that the venous occlusion-rebound component also constitutes a novel cerebral venous preconditioning (CVPC) modality that, unlike arterial-based ischemic preconditioning, whether local or remote (e.g., remote ischemic preconditioning, RIPC) [6], acts directly on the cerebral circulation. As a further, more speculative extension addressed later in this report, this direct route may also engage central neuroendocrine and autonomic regulatory structures otherwise inaccessible via any remote conditioning modality.

The glymphatic system is a perivascular network through which CSF enters the brain via periarterial spaces, exchanges with interstitial fluid, and exits via perivenous spaces, carrying soluble metabolic waste products, including amyloid-beta and tau. This clearance process depends substantially on the pressure gradients and pulsatile flow driving CSF movement through the perivascular network [7]. Given this dependence on CSF pressure dynamics and cerebral venous outflow, mechanical interventions that transiently alter intracranial pressure gradients or venous drainage patterns may plausibly influence glymphatic clearance efficiency, providing the physiological rationale for the second hypothesis explored in this report.

Both neurodegenerative and spinal degenerative diseases often begin decades before clinical symptoms appear. In autosomal dominant Alzheimer's disease, amyloid processing changes may begin up to 25 years before expected symptom onset, with measurable amyloid deposition detectable at least 15 years prior [8]. In the author's view, this subclinical timeline offers a conceptual, rather than an evidenced, parallel to spinal degeneration: chronic spinal compression similarly contributes to disc degeneration, osteophyte formation, and spinal stenosis long before pain or neurological deficits develop. No causal or epidemiological evidence currently links these two processes, and this analogy is not intended to equate their evidentiary strength or clinical urgency; rather, it is offered as a motivating framework for the report's dual-hypothesis structure, addressing spinal and glymphatic subclinical progression as parallel, independently justified targets for a single preventive intervention. ACT is proposed here as a preventive biomechanical intervention addressing both processes simultaneously. Through daily self-administered practice, ACT directly targets posture correction and spinal decompression while potentially enhancing intracranial circulation, augmenting glymphatic clearance, and delivering CVPC.

## Technical report

Active craniospinal tensioning (ACT)

**Figure 1:**
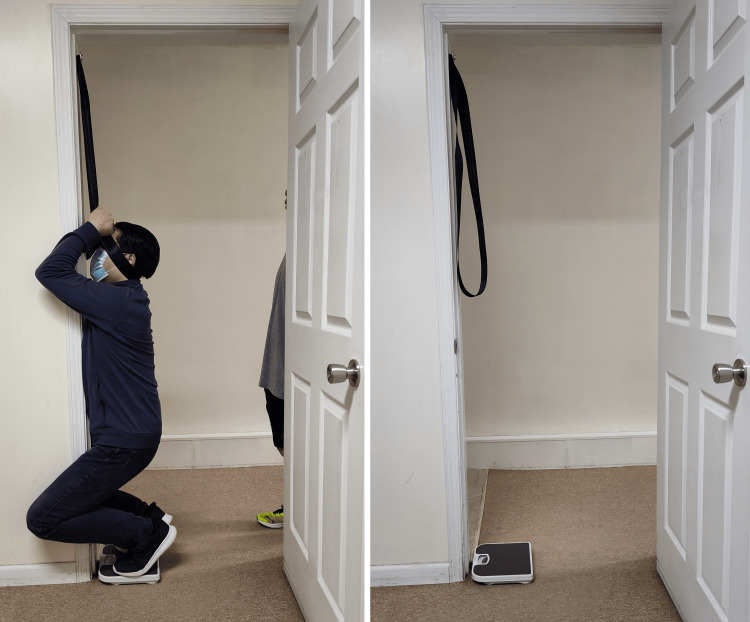
Active craniospinal tensioning (ACT): technique demonstration A 48-50 mm wide nylon or polyester webbing strap is routed from beneath the external occipital protuberance anteriorly and superiorly, passing over the lower ear, primarily the earlobe, and clasped at the forehead. The hands close the loop only as a passive fail-safe and do not contribute to the tensile force. ACT is generated by a voluntary partial squat, transmitting sub-waist body weight upward through the spine to the occipital anchor. Maximum transmissible load approximates 50% of body weight at the lumbosacral junction and 80% at the occipitoatlantal junction, representing total body weight minus the head and the strap-borne portion. In practice, transmitted load is substantially lower than this theoretical maximum. A spring-loaded scale under both feet quantifies residual floor-borne weight during full relaxation, serving as a biofeedback measure of relaxation depth. For the author (175 lb), the lowest residual weight achieved was approximately 40 lb, corresponding to an estimated distraction load of approximately 100 lb at the occipitoatlantal junction. Each session consists of a single two- to five-second hold, performed one to three times daily. The lower bound reflects the minimum duration for tensile force to fully transmit through the spine [3]; the upper bound reflects the typical onset of mild presyncope, at or near which most clinical and home practice occurs, provided a safety spotter is present (see main text). Duration and frequency may be adjusted at the discretion of the clinician based on individual response and tolerance. ACT should be initiated only after clinical instruction and screening, with a safety spotter present throughout. Image credit: Author (as participant) and consenting model (as safety spotter)

ACT is a self-administered technique whose procedural configuration, equipment requirements, biomechanical implementation, safety parameters, contraindications, and precautions have been described in a prior technical report [3]. Following appropriate screening and clinical instruction, the maneuver can be performed independently by mobile ambulatory participants (Figure 1).

ACT presyncope phenomenon

The author reports relaxing the entire body while maintaining grip on the strap. In the author's self-observation, within five seconds, vision begins to dim; by 10 seconds, dimming intensifies and high-pitched tinnitus develops. The point at which unconsciousness would occur has not been formally tested and remains unknown; this uncertainty is emphasized rather than minimized. The maneuver is terminated well before any anticipated loss of consciousness, at the first onset of presyncopal symptoms. Loss of consciousness during a self-administered technique anchored over the occiput poses a risk of fall; this technique should not be attempted without prior instruction from a trained clinician, and no attempt should be made to test or approach a loss-of-consciousness threshold.

The presyncope phenomenon associated with ACT appears to depend on posture-specific factors rather than on suboccipital compression alone. The author's previously described pelvis-stabilized axial spinal traction (PSAST) technique [5] applies axial spinal traction of comparable force and duration to ACT but from a supine position and similarly involves suboccipital compression; however, presyncope has not been observed under these conditions. The mechanism underlying this postural difference is addressed below.

The presyncope resolves within approximately two seconds after ACT release, based on the author's self-monitored timing and informal clinical observations across multiple patients. These timing estimates are anecdotal, have not been systematically or prospectively collected, and are not supported by instrumented physiological measurements. Resolution is typically accompanied by a transient sensation of warmth and cranial fullness, consistent with the reactive arterial reperfusion mechanism described below. The presyncope response is hypothesized, rather than demonstrated, to represent a potentially beneficial physiological stress, not solely an adverse effect warranting caution. This interpretation remains speculative, is discussed further below, and should not be construed as an empirically established therapeutic effect.

ACT for posture correction and spinal decompression

The intervertebral disc exhibits both elastic and viscoelastic mechanical responses to loading. When a compressive load is applied, the disc immediately deforms (the elastic response) and then slowly continues to deform over subsequent minutes and hours as fluid is expressed from the nucleus pulposus (the viscoelastic response) [9]. The reverse applies under tensile force: immediate elastic decompression occurs, transiently reducing intradiscal pressure in proportion to the applied load. This pressure reduction is force-dependent rather than duration-dependent [10]. The brief duration of ACT, therefore, primarily produces elastic disc decompression with each application.

A useful reference for the general scale of spinal elongation the body can undergo is the well-documented diurnal height loss of approximately 19.3 mm, or about 1.1% of overall stature [11], though this figure reflects a mechanistically distinct process, the slow viscoelastic expression of fluid from the intervertebral discs under sustained gravitational loading throughout the waking day [12], rather than the rapid elastic decompression ACT is proposed to produce. ACT generates transmissible tensile loads at the occipitoatlantal junction that substantially exceed the gravitational compressive load the head normally imposes on the cervical spine, with the precise magnitude depending on the participant's squat technique and body mechanics during the maneuver. It is hypothesized that this high-force, brief distraction can mechanically produce an immediate elastic elongation, though whether its magnitude approaches that of diurnal viscoelastic recovery remains untested and is not assumed here.

Decades of sustained gravitational compression can lead to progressive spinal degeneration and excessive structural curvature. The hyperkyphotic configuration is typically characterized by an exaggerated thoracic curve, anterior head carriage, and reduced thoracic volume secondary to chronic axial loading. ACT is hypothesized to counteract these degenerative changes by reducing hyperkyphotic curvature, restoring optimal cervical and sagittal alignment, and expanding thoracic volume through high-force axial spinal elongation. This theoretical mechanical model presents a targeted approach to global postural realignment, though formal objective postural assessments, spinal imaging, and spirometric measurements are required to confirm these specific structural outcomes.

Future investigation of ACT's structural and physiological effects would benefit from instrumented measurement rather than self-observation. Candidate methods include intradiscal pressure monitoring during the maneuver and pre- and post-ACT stadiometry to quantify changes in acute elastic height. These measurements fall outside the scope of the present technical report and are identified here as priorities for future controlled study.

Biomechanical rationale for accelerated glymphatic clearance

Metabolic waste products progressively accumulate in the brain as glymphatic clearance efficiency declines with age and disease burden. Under baseline conditions, glymphatic transport is driven primarily by three physiological forces: cardiac arterial pulsation, arteriolar vasomotion, and respiratory oscillation [7], each contributing convective perivascular fluid movement that, while adequate under healthy conditions, may become insufficient as interstitial protein burden increases.

It is hypothesized that the proposed ACT protocol's two coordinated components, dural pull-recoil and suboccipital venous occlusion-rebound, augment convective perivascular clearance, thereby theoretically enhancing bulk exchange of CSF and interstitial fluid beyond what baseline physiological drivers alone can sustain.

Dural Pull-Recoil Mechanism

The potential role of axial spinal traction in modulating CSF and glymphatic circulation through a dural pull-recoil mechanism has been proposed previously [3,5]. Briefly, the craniospinal compartment comprises the intracranial dura mater and its caudal continuation, the spinal dural sac, forming a continuous hydraulic system through which CSF flows across the foramen magnum. The spinal dura mater attaches superiorly near the foramen magnum and inferiorly at the sacrum, with the intervening segment retaining limited longitudinal distensibility. The proposed mechanism is as follows: ACT transiently elongates the vertebral column and tensions the spinal dural sac, generating a transient pressure gradient within the craniospinal compartment; the traction and release phases propagate this gradient across the foramen magnum, resulting in CSF displacement; this CSF agitation in turn disturbs the perivascular and interstitial fluid compartments, suspending soluble neurotoxic metabolites, including amyloid-beta and tau, within the perivascular flow stream and thereby making them more available for convective clearance via the glymphatic efflux pathway. No direct measurements of CSF pressure, flow, or metabolite clearance during ACT have been performed; this entire mechanistic sequence remains an untested hypothesis pending empirical validation.

Suboccipital Venous Occlusion and the Five-Second Safety Ceiling

The shift to an upright position alters the gravitational dynamics of cerebral venous return, favoring drainage through the suboccipital venous plexus, here defined as encompassing the local vertebral venous plexus and posterior emissary channels, over the internal jugular route [13]. The anatomical routing of the ACT harness strap beneath the external occipital protuberance and across the suboccipital triangle is likely to exert an evenly distributed, blunt compressive force upon the suboccipital venous plexus. This local compression creates a brief, bilateral bottleneck for venous blood exiting the posterior cranial fossa. As arterial inflow continues through the internal carotid and vertebral arteries, the latter anatomically protected from strap pressure by their deep cervical positions within the transverse foramina, the trapped venous volume accumulates within the dural sinuses, propagating pressure retrograde into the cerebral capillary beds and generating a transient rise in intracranial venous pressure.

Beyond the five-second mark, continued venous outflow restriction results in progressive venous hypertension, elevating intracranial pressure (ICP) and thereby reducing cerebral perfusion pressure (CPP) [14]. As CPP approaches the lower limit of cerebral autoregulation, reduced cerebral and retinal perfusion may manifest as visual dimming and high-pitched tinnitus. The visual dimming may reflect reduced perfusion of the retina and visual cortex [15], and the tinnitus likely reflects a transient increase in pressure within the transverse sinus [16].

It is hypothesized that these symptoms serve as a real-time functional biomarker of the maneuver's intracranial efficacy rather than generic adverse events, consistent with the idea that suboccipital compression has successfully occluded intracranial venous return and generated localized venous hypertension within the transverse and sagittal sinuses. Their onset represents an operational physiological ceiling, and the brief, high-pressure bottleneck they signal is hypothesized to act as a hormetic stressor, elastic-loading the intracranial hydraulic architecture with the potential energy required to drive the subsequent rebound phase upon release.

The five-second ceiling is not a published physiological threshold but an empirically derived safety boundary. It is not a limit to remain under, but the point around which mild presyncopal onset (vision dimming) is expected and considered acceptable, provided a safety spotter is present. Mild presyncope is hypothesized to be the perceptual signature that venous occlusion has reached a sufficient threshold to drive the subsequent rebound phase, and its absence likely indicates that occlusion was not achieved. The five-second figure reflects sessions with adequate relaxation depth; participants who do not achieve comparable relaxation may not reach presyncopal threshold even after 10 seconds or longer, consistent with the floor-scale biofeedback measure of relaxation depth described above. The maneuver should be terminated promptly upon perceiving vision dimming, allowing the brief reaction interval inherent in self-perception, rather than allowing symptoms to intensify or tinnitus to develop, as these indicate that the individual's perfusion threshold has been substantially exceeded.

An additional anatomical fail-safe limits the accumulation of intracranial venous pressure independently of participant behavior. In the normal upright posture, the internal jugular veins remain collapsed, as cerebral venous drainage preferentially flows through the suboccipital venous plexus. However, jugular collapse is a dynamic pressure equilibrium rather than a fixed anatomical state: as intracranial venous pressure rises during ACT tensioning, the upstream pressure gradient eventually exceeds the jugular collapse threshold, recruiting the internal jugular veins as an additional drainage pathway that caps further pressure accumulation [17]. This overflow mechanism, closed under normal upright conditions, opens automatically at a critical pressure threshold and self-limits the maximum achievable intracranial venous pressure.

The ACT safety architecture thus comprises four independent and redundant layers: the behavioral five-second ceiling, the perceptual presyncope dosimetry signals, the hand-hold fail-safe upon syncope, and the anatomical jugular overflow mechanism, the latter two of which require no conscious participant action and remain operative even in the event of compliance failure.

The Post-Release Hemodynamic Rebound and Proposed Glymphatic Clearance Mechanism

It is hypothesized that upon abrupt release of suboccipital compression, the congested dural sinuses rapidly decompress, producing a sudden surge in antegrade venous outflow. This rebound is proposed to directly address the efflux side of glymphatic clearance, actively promoting the removal of metabolic waste accumulated within the brain parenchyma. As noted above, this hemodynamic recovery is accompanied by a transient sensation of warmth and cranial fullness, consistent with reactive arterial reperfusion delivering oxygenated blood to previously underperfused tissue.

It is hypothesized that the two ACT mechanisms operate in rapid, simultaneous coordination to constitute a complete glymphatic clearance cycle. The dural pull-recoil component, described above, suspends soluble macromolecules, including amyloid-beta and tau, within the perivascular flow stream. Concurrently, the venous occlusion-rebound component drives their removal: the powerful rebound accelerates perivenous efflux, the primary drainage arm of the glymphatic system, clearing the suspended metabolic load from the cerebral interstitium. Together, pressure-driven agitation and accelerated efflux function as coupled phases of a single mechanical event, acting on both the suspension and the drainage of soluble protein aggregates in a single coordinated cycle.

## Discussion

Suboccipital venous occlusion-rebound as a potential cerebral venous preconditioning (CVPC) mechanism

Ischemic preconditioning, whether applied locally at the target organ or remotely at a limb (e.g., RIPC), achieved through brief, repeated cycles of arterial occlusion and release, confers protection against ischemia-reperfusion injury across multiple organ systems, with documented cardioprotective, neuroprotective, and renoprotective effects mediated through neurogenic, humoral, and antioxidant pathways [6]. Local arterial preconditioning, though mechanistically direct, is not deliberately performed in the human brain, as repeated cerebral arterial occlusion for preconditioning purposes carries unacceptable ischemic risk and is not ethically permitted outside incidental clinical contexts such as transient ischemic attacks or intraoperative arterial clamping. Specifically in cerebrovascular applications, RIPC has demonstrated stroke-protective effects in clinical trials, reducing the recurrence of intracranial arterial stenosis [18] and periprocedural stroke during carotid stenting [19]. Its protective mechanism operates through endogenous ischemic preconditioning pathways: upregulation of heat shock proteins, reduction of inflammatory adhesion molecules, endothelial conditioning, and neuronal preparation for tolerance of subsequent ischemic insults. A standard RIPC session requires four to five cycles of five minutes on and five minutes off, totaling approximately 40-50 minutes per session. This clinical evidence base for RIPC, comprising multiple randomized controlled trials and a well-defined dosing protocol, has no counterpart for ACT; no controlled trial data currently exist evaluating venous occlusion-rebound as a preconditioning stimulus. The comparison drawn below is therefore offered explicitly as a hypothesis to motivate future investigation, not as evidence of comparable efficacy or of equivalent evidentiary standing.

Ischemic preconditioning was first demonstrated in myocardial tissue in 1986 [20], and the subsequent four decades of research have established local preconditioning as the mechanistically most direct route to ischemic tolerance, acting on the target tissue itself without reliance on humoral or neural relay; yet for the brain specifically, this superiority has remained clinically inaccessible, as local arterial preconditioning carries unacceptable ischemic risk when applied to cerebral tissue. Suboccipital venous occlusion during the tensioning phase, followed by rapid pressure release upon recoil, is a direct biomechanical consequence of the maneuver. It is hypothesized that this occlusion-rebound event may function as a novel modality for cerebral venous preconditioning (CVPC): engaging endogenous preconditioning pathways analogous to those invoked by existing arterial occlusion-rebound modalities [6], but applying them directly to the cerebral circulation, at a site and via a route inaccessible to any existing modality, and within a markedly safer physiological range than arterial occlusion would permit. Beyond this preconditioning effect, CVPC is hypothesized to act in concert with the glymphatic clearance mechanism described in the Technical Report, a synergy addressed in the Conclusions; the potential downstream engagement of central neuroendocrine and autonomic pathways is likewise discussed as a speculative extension.

This proposed direct-tissue delivery route is offered as a rationale for future study and is not presented as evidence that ACT achieves preconditioning effects comparable to RIPC, nor that a brief stimulus duration is functionally equivalent to RIPC's established dosing protocol; no dose-response relationship for CVPC has been established.

Traction harness design rationale

In the author's retrospective clinical observations spanning more than eight years of ambulatory ACT practice, a consistent variance in vascular responsiveness has been observed across participants. A subset reaches the hemodynamic threshold and experiences transient, self-limiting presyncope, characterized by visual dimming and, if the hold continues, high-pitched tinnitus, within the traction window, while the remaining participants report predominantly musculoskeletal decompression with few neurovascular symptoms. This variance is hypothesized to reflect two contributors: anatomical differences in suboccipital venous plexus density and incomplete axial load transfer, most commonly due to insufficient participant relaxation, in which residual lower-limb muscular activity partially offloads body weight through foot contact with the floor, reducing the proportion of transmissible load directed to the occipital anchor. A spring-loaded scale placed underfoot helps quantify how much weight is being offloaded onto the ground (Figure 1) for training purposes.

It is hypothesized that the venous escape inherent in a flat textile strap represents a functional design equilibrium rather than a confounding limitation, serving a protective and therapeutic role in the maneuver. Because ACT is a dual-mechanism intervention, venous occlusion and axial spinal traction must operate within the same hold duration. A perfectly sealing interface that eliminates venous escape entirely would trigger presyncope so rapidly that the axial traction window, and with it the posture correction and spinal decompression benefits, would be substantially curtailed. The flat strap's incomplete occlusion allows sufficient venous pressure cycling to augment glymphatic efflux while preserving a five-second hold long enough for full spinal decompression and postural correction. Cost-effectiveness and universal accessibility further support the flat textile strap as the current optimal configuration for this dual-mechanism application.

The falsifiable endpoint for ACT's proposed glymphatic effect

The central testable prediction is twofold: that a single ACT session acutely elevates DTI-ALPS above resting baseline, and that daily practice sustained for three to six months, followed by a repeat measurement, produces a measurable upward shift in baseline glymphatic indices relative to the pre-practice value. DTI-ALPS is the most widely adopted non-invasive surrogate marker for glymphatic function currently available [4]. While its specificity for perivascular flow remains under active methodological scrutiny, it represents the best available falsifiable endpoint for the hypothesis.

The author proposes a two-tier design, expanding on the protocol briefly outlined in the ACT technical report [3].

Tier 1 (single session) addresses the acute mechanistic question through a within-subject pre/post design: a baseline DTI-ALPS acquisition, a single ACT maneuver, and an immediate post-maneuver acquisition. The within-subject structure eliminates the dominant source of variance in ALPS measurement, individual differences in perivascular architecture, and the index is sufficiently removed from conscious physiological control that expectation effects are not a credible alternative explanation for a positive result. A null result warrants adjusted dosing parameters, a repeated-maneuver protocol, or a delayed acquisition window before the hypothesis is formally disconfirmed. An ideal candidate is one who reliably achieves presyncopal symptoms within the target hold duration, for example, within five seconds, serving as a physiological indicator of sufficient tensile load delivery.

Tier 2 (daily practice, three to six months) addresses the clinically relevant question of whether sustained ACT practice shifts baseline glymphatic function over time. A pre-/post-design comparing DTI-ALPS at baseline and after three to six months of daily practice would test this directly. Secondary endpoints should include sleep quality indices and, where feasible, measures of cognitive function and visual acuity, domains in which subjective improvements are frequently reported in the author's clinical observations. A positive result in an already-affected population, particularly attenuation of the longitudinal DTI-ALPS decline that characterizes neurodegenerative disease progression, would provide the first direct empirical support for a disease-modifying effect of ACT, and would in turn motivate a separate trial tier in at-risk, pre-symptomatic populations to test the distinct claim that daily home practice could delay or prevent disease onset.

## Conclusions

This report addresses two mechanistically related claims, presented at different levels of evidentiary support, alongside a framework for future speculative research.

First, regarding the established biomechanical basis that requires further confirmation: the structural claim that daily ACT practice may produce cumulative benefits for posture correction and spinal decompression is supported by established spinal biomechanics but requires formal confirmation through objective postural assessment, spirometry, and spinal imaging.

Second, regarding the original theoretical synthesis proposed across this report and the author's prior technical reports: the fluid-dynamic claim, that the two integrated components of ACT, dural pull-recoil and suboccipital venous occlusion-rebound, converge on cerebral perivenous outflow conditions in a manner consistent with augmented glymphatic drainage, remains at the hypothesis stage and awaits its first direct test via DTI-ALPS, as proposed here, and constitutes an original theoretical contribution establishing a novel conceptual framework for targeting glymphatic drainage. Independently of this glymphatic hypothesis, suboccipital venous occlusion-rebound is separately proposed as a distinct, venous-side alternative to traditional arterial-based ischemic preconditioning, introducing CVPC as a mechanism-level original contribution that does not depend on confirmation of the glymphatic clearance hypothesis, though their combined, rather than isolated, contribution to functional neuroprotective outcomes is addressed below.

Third, and most speculatively, the potential systemic implications of CVPC extend beyond localized cerebral fluid dynamics and are not themselves supported by evidence presented in this report. It is further hypothesized that CVPC's neuroprotective value may be functionally contingent on, rather than independent of, effective glymphatic clearance: ischemic tolerance conferred by venous-side preconditioning may confer limited benefit to brain tissue if metabolic waste and toxic protein aggregates are not concurrently removed from the perivascular space, since accumulated clearance failure is itself a driver of tissue dysfunction regardless of the tissue's preconditioned state. Under this view, CVPC and glymphatic augmentation are proposed as synergistic rather than independently sufficient mechanisms, even though each remains separately falsifiable as established in the preceding paragraph. As a further speculative extension, noted in the Discussion, whether direct venous-side conditioning can engage central neuroendocrine and brainstem autonomic centers to deliver downstream cardiovascular, cerebrovascular, or organ-protective benefits is identified solely to motivate independent future investigation alongside the primary glymphatic endpoint.

## References

[1] Hansraj KK (2014). Assessment of stresses in the cervical spine caused by posture and position of the head. Surg Technol Int.

[2] Margetis K, Singh C, Casiano VE (2026). Low back pain: evaluation and management. https://www.ncbi.nlm.nih.gov/books/NBK538173/.

[3] Chen HW (2026). Active craniospinal tensioning (ACT): axial spinal traction for glymphatic modulation. Cureus.

[4] Taoka T, Ito R, Nakamichi R, Nakane T, Kawai H, Naganawa S (2024). Diffusion tensor image analysis ALong the perivascular space (DTI-ALPS): revisiting the meaning and significance of the method. Magn Reson Med Sci.

[5] Chen HW (2025). Axial spinal traction as a potential modulator of cerebrospinal and glymphatic circulation in neurodegenerative diseases: a technical report and biomechanical hypothesis. Cureus.

[6] Hao Y, Xin M, Feng L, Wang X, Wang X, Ma D, Feng J (2020). Review cerebral ischemic tolerance and preconditioning: methods, mechanisms, clinical applications, and challenges. Front Neurol.

[7] Rasmussen MK, Mestre H, Nedergaard M (2022). Fluid transport in the brain. Physiol Rev.

[8] Bateman RJ, Xiong C, Benzinger TL (2012). Clinical and biomarker changes in dominantly inherited Alzheimer's disease. N Engl J Med.

[9] O'Connell GD, Jacobs NT, Sen S, Vresilovic EJ, Elliott DM (2011). Axial creep loading and unloaded recovery of the human intervertebral disc and the effect of degeneration. J Mech Behav Biomed Mater.

[10] Han C, Feng M, Wen H (2024). Rotation-traction manipulation induced intradiskal pressure changes in cervical spine-an in vitro study. Front Bioeng Biotechnol.

[11] Reilly T, Tyrrell A, Troup JD (1984). Circadian variation in human stature. Chronobiol Int.

[12] Adams MA, Dolan P, Hutton WC, Porter RW (1990). Diurnal changes in spinal mechanics and their clinical significance. J Bone Joint Surg Br.

[13] San Millán Ruíz D, Gailloud P, Rüfenacht DA, Delavelle J, Henry F, Fasel JH (2002). The craniocervical venous system in relation to cerebral venous drainage. AJNR Am J Neuroradiol.

[14] Wilson MH (2016). Monro-Kellie 2.0: the dynamic vascular and venous pathophysiological components of intracranial pressure. J Cereb Blood Flow Metab.

[15] Dattilo M, Newman NJ, Biousse V (2018). Acute retinal arterial ischemia. Ann Eye Sci.

[16] Chiarella G, Bono F, Cassandro C, Lopolito M, Quattrone A, Cassandro E (2012). Bilateral transverse sinus stenosis in patients with tinnitus. Acta Otorhinolaryngol Ital.

[17] Gisolf J, van Lieshout JJ, van Heusden K, Pott F, Stok WJ, Karemaker JM (2004). Human cerebral venous outflow pathway depends on posture and central venous pressure. J Physiol.

[18] Meng R, Asmaro K, Meng L (2012). Upper limb ischemic preconditioning prevents recurrent stroke in intracranial arterial stenosis. Neurology.

[19] Zhao W, Meng R, Ma C (2017). Safety and efficacy of remote ischemic preconditioning in patients with severe carotid artery stenosis before carotid artery stenting: a proof-of-concept, randomized controlled trial. Circulation.

[20] Murry CE, Jennings RB, Reimer KA (1986). Preconditioning with ischemia: a delay of lethal cell injury in ischemic myocardium. Circulation.

